# Complete genome sequence of *Pectobacterium brasiliense* strain 21PCA_AGRO2 with antimicrobial resistance isolated from napa cabbage

**DOI:** 10.1128/MRA.00066-23

**Published:** 2023-09-07

**Authors:** Sojin Ahn, So Yun Jhang, Eunbyeol Ahn, Sangryeol Ryu, Jaewoong Yu

**Affiliations:** 1 eGnome Inc., Seoul, Republic of Korea; 2 Interdisciplinary Program in Bioinformatics, Seoul National University, Seoul, Republic of Korea; 3 Department of Food and Animal Biotechnology, Department of Agricultural Biotechnology and Research Institute of Agriculture and Life Sciences, Seoul National University, Seoul, Republic of Korea; University of Maryland School of Medicine, Baltimore, Maryland, USA

**Keywords:** whole-genome sequence, complete genome, *Pectobacterium*, Nanopore sequencing

## Abstract

We report a complete genome of *Pectobacterium brasiliense* strain 21PCA_AGRO2 isolated from napa cabbage, in which the genome consists of a circular chromosome comprising 4,919,671 bp with 4,399 coding DNA sequences, 22 rRNA genes, 77 tRNA genes, and 9 noncoding RNA genes.

## ANNOUNCEMENT

The genus *Pectobacterium* is a plant pathogen that causes soft rot on a variety of economically important crops, such as potatoes, napa cabbages, and radishes (1). Long-term use of agricultural antibiotics to control the soft rot may lead to problems with antibiotic resistance (2, 3). Thus, we conducted whole-genome sequencing of *P. brasiliense* 21PCA_AGRO2 isolated from napa cabbage. This will provide insight into the pathogenic bacterial genome and promote further research to track the antibiotic resistance in agricultural products.

*P. brasiliense* 21PCA_AGRO2 was isolated from soft rot lesion in napa cabbage collected from Pyeongchang, South Korea. The infected plant tissues were sterilized with 1% hypochlorite solution for 90 s, rinsed in double distilled water, and then ground into homogenate with 1 mM MgSO_4_ by stomacher (BagMixer 400 Laboratory Blender, Interscience, UK). The single colony was cultured at 28°C in LB (Luria-Bertani) media with overnight, diluted 1:100 with fresh LB broth, and then incubated 18–20 h at 28°C with shaking at 200 rpm. Colonies on plates were picked and confirmed with PCR using specific primers for pectate lyase (*pel*) genes (5′-TTACCGGACGCCGAGCTGTGGCGT-3′ and 5′-CAGGAAGATGTCGTTATCGCGAGT-3′) of *Pectobacterium* spp. and 16S rRNA (5′-AGAGTTTGATCCTGGCTCAG-3′ and 5′-GGTTACCTTGTTACGACTT-3′) sequencing. The 16S rRNA matched more than 99.5% with the reference sequences of *P. brasiliense* in BLAST database. Afterward, genomic DNA (gDNA) was extracted using Kit PureHelix Genomic DNA Prep Kit (solution type)-Bacteria and quantified and qualified by Quant-iT PicoGreen dsDNA Assay Kit (Invitrogen, USA). The Short Read Eliminator kit was used to remove <10 kbp fragments from unsheared gDNA. The Oxford Nanopore Technologies sequencing library was prepared using the manufacturer’s ligation sequencing kit (SQK-LSK 112, UK) and sequenced on a MinION MK1b device (MIN112, R10.4) with MinKNOW software (22.05.5).

**Fig 1:**
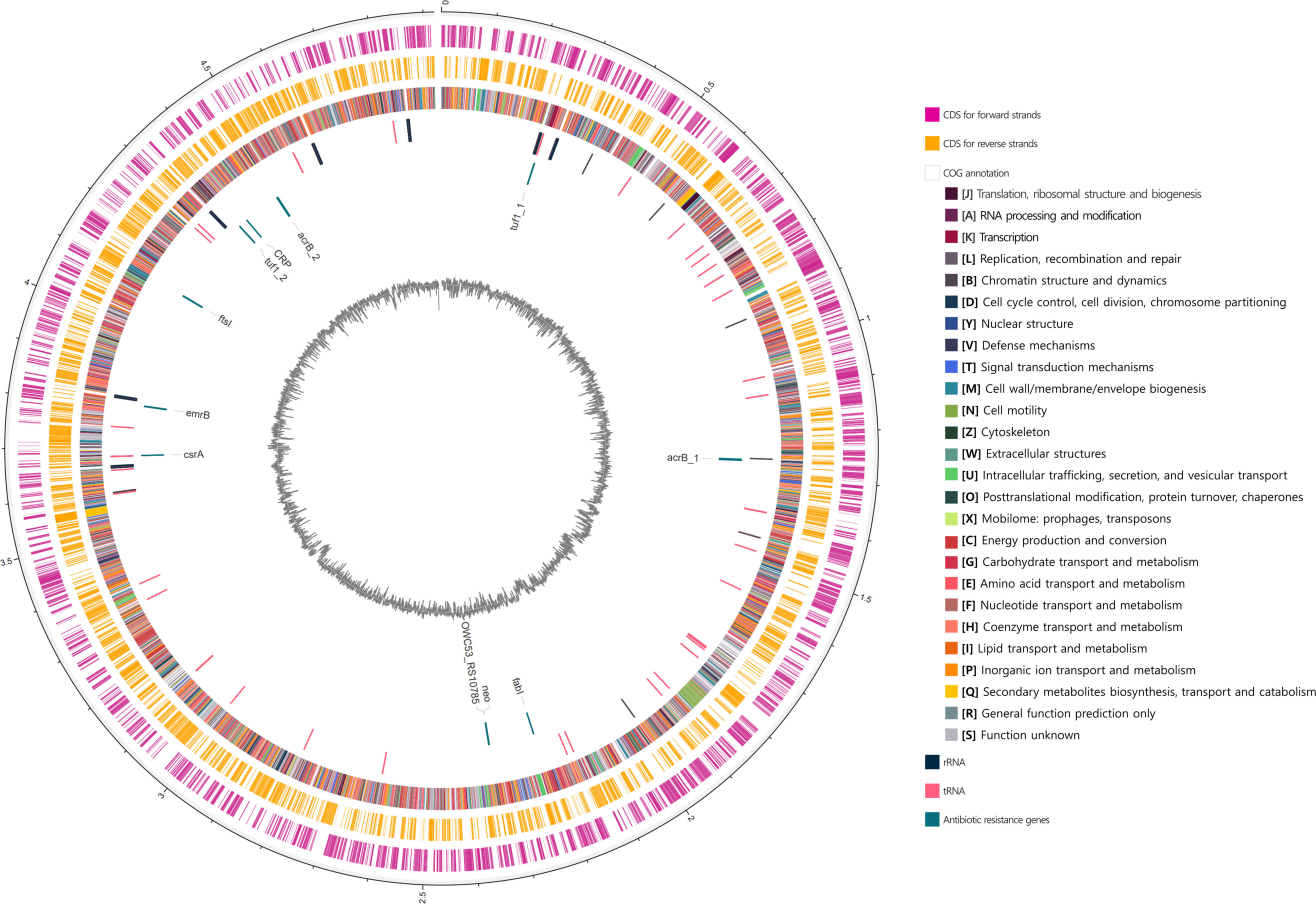
The genome map of *Pectobacterium brasiliense* strain 21PCA_AGRO2. Each circle indicates coding sequences (CDS) in the leading strand, CDS in the lagging strand, COG distribution, RNA, antibiotic resistance genes, and the GC contents from outer to inner. Antibiotic resistance genes are labeled.

A total of 64,110 raw reads (*N*_50_ 25,167 bp) were produced and then trimmed and quality filtered using Porechop v2.0.4 (https://github.com/rrwick/Porechop). The *de novo* assembly was conducted using flye v2.9-b1778 and confirmed genome completeness using BUSCO v5.2.2 (4, 5). Next, the genome was rotated using *dnaA* as the start position based on the fixstart method in Circlator v1.5.5 (6) and annotated with Prokaryotic Genome Annotation Pipeline (PGAP) v6.5 (7). As a result, the complete genome sequence comprised one circular form of 4,919,671 bp and a GC content of 51.68%, yielding 4,399 coding sequences (including 28 frameshifted genes), 22 rRNAs, 77 tRNAs, and 9 ncRNAs (Fig. 1). Subsequently, taxonomic classification was performed using GTDB-tk v1.5.1 (8) and Kraken2 v2.1.2 (9), which is identified as *P. brasiliense*. The average nucleotide identity (ANI) analysis was performed using FastANI v1.0 (10) to compare our genome with 31 species of published *P. carotovorum* and *P. brasiliense* genome sequences. As a consequence, our genome was closest to the *P. brasiliense* ZLMLSHJ5 strain (GCF_016864975.1) with an ANI value of 97.55%. Additionally, antibiotic resistance gene prediction using RGI v5.2.1 against CARD database identified 11 antibiotic resistance genes, including aminoglycoside resistance (11). Moreover, eggNOG-mapper v2.1.6 (12) was used with the eggNOG v5 (13) database to assign the Clusters of Orthologous Groups (COGs) functional categories based on biological systems. All the bioinformatics tools were used with default options unless specified otherwise.

## Data Availability

The whole genome sequence was deposited in GenBank under the accession number CP113504, BioProject accession number PRJNA906323 and SRA accession number SRR22542065.

## References

[1] Oulghazi S, Sarfraz S, Zaczek-Moczydłowska MA, Khayi S, Ed-Dra A, Lekbach Y, Campbell K, Novungayo Moleleki L, O’Hanlon R, Faure D. 2021. Pectobacterium brasiliense: genomics, host range and disease management. Microorganisms 9:106. doi:10.3390/microorganisms9010106 33466309 PMC7824751

[2] Czajkowski R, Pérombelon MCM, van Veen JA, van der Wolf JM. 2011. Control of blackleg and tuber soft rot of potato caused by Pectobacterium and Dickeya species: a review. Plant Pathol 60:999–1013. doi:10.1111/j.1365-3059.2011.02470.x

[3] Charkowski AO, Lind J, Rubio-Salazar I. 2014. Genomics of plant-associated bacteria: the soft rot Enterobacteriaceae. Genomics of plant-associated bacteria:37–58. doi:10.1007/978-3-642-55378-3

[4] Kolmogorov M, Yuan J, Lin Y, Pevzner PA. 2019. Assembly of long, error-prone reads using repeat graphs. Nat Biotechnol 37:540–546. doi:10.1038/s41587-019-0072-8 30936562

[5] Manni M, Berkeley MR, Seppey M, Simão FA, Zdobnov EM. 2021. BUSCO update: novel and streamlined workflows along with broader and deeper phylogenetic coverage for scoring of eukaryotic, prokaryotic, and viral genomes. Mol Biol Evol 38:4647–4654. doi:10.1093/molbev/msab199 34320186 PMC8476166

[6] Hunt M, Silva ND, Otto TD, Parkhill J, Keane JA, Harris SR. 2015. Circlator: automated circularization of genome assemblies using long sequencing reads. Genome Biol 16:294. doi:10.1186/s13059-015-0849-0 26714481 PMC4699355

[7] Li W, O’Neill KR, Haft DH, DiCuccio M, Chetvernin V, Badretdin A, Coulouris G, Chitsaz F, Derbyshire MK, Durkin AS, Gonzales NR, Gwadz M, Lanczycki CJ, Song JS, Thanki N, Wang J, Yamashita RA, Yang M, Zheng C, Marchler-Bauer A, Thibaud-Nissen F. 2021. RefSeq: expanding the prokaryotic genome annotation pipeline reach with protein family model curation. Nucleic Acids Res 49:D1020–D1028. doi:10.1093/nar/gkaa1105 33270901 PMC7779008

[8] Chaumeil P-A, Mussig AJ, Hugenholtz P, Parks DH. 2019. GTDB-TK: a toolkit to classify genomes with the genome taxonomy database. Bioinformatics 36:1925–1927. doi:10.1093/bioinformatics/btz848 31730192 PMC7703759

[9] Wood DE, Lu J, Langmead B. 2019. Improved metagenomic analysis with Kraken 2. Genome Biol 20:257. doi:10.1186/s13059-019-1891-0 31779668 PMC6883579

[10] Jain C, Rodriguez-R LM, Phillippy AM, Konstantinidis KT, Aluru S. 2018. High throughput ANI analysis of 90K prokaryotic genomes reveals clear species boundaries. Nat Commun 9:5114. doi:10.1038/s41467-018-07641-9 30504855 PMC6269478

[11] Alcock BP, Raphenya AR, Lau TTY, Tsang KK, Bouchard M, Edalatmand A, Huynh W, Nguyen A-L, Cheng AA, Liu S, Min SY, Miroshnichenko A, Tran H-K, Werfalli RE, Nasir JA, Oloni M, Speicher DJ, Florescu A, Singh B, Faltyn M, Hernandez-Koutoucheva A, Sharma AN, Bordeleau E, Pawlowski AC, Zubyk HL, Dooley D, Griffiths E, Maguire F, Winsor GL, Beiko RG, Brinkman FSL, Hsiao WWL, Domselaar GV, McArthur AG. 2020. CARD 2020: antibiotic resistome surveillance with the comprehensive antibiotic resistance database. Nucleic Acids Res 48:D517–D525. doi:10.1093/nar/gkz935 31665441 PMC7145624

[12] Cantalapiedra CP, Hernández-Plaza A, Letunic I, Bork P, Huerta-Cepas J. 2021. eggNOG-mapper v2: functional annotation, orthology assignments, and domain prediction at the metagenomic scale. Mol Biol Evol 38:5825–5829. doi:10.1093/molbev/msab293 34597405 PMC8662613

[13] Huerta-Cepas J, Szklarczyk D, Heller D, Hernández-Plaza A, Forslund SK, Cook H, Mende DR, Letunic I, Rattei T, Jensen LJ, von Mering C, Bork P. 2019. eggNOG 5.0: a hierarchical, functionally and phylogenetically annotated orthology resource based on 5090 organisms and 2502 viruses. Nucleic Acids Res 47:D309–D314. doi:10.1093/nar/gky1085 30418610 PMC6324079

